# Advancements in the Application of the Fenton Reaction in the Cancer Microenvironment

**DOI:** 10.3390/pharmaceutics15092337

**Published:** 2023-09-18

**Authors:** Rile Ou, Gerile Aodeng, Jun Ai

**Affiliations:** Inner Mongolia Key Laboratory of Environmental Chemistry, College of Chemistry and Enviromental Science, Inner Mongolia Normal University, 81 Zhaowudalu, Hohhot 010022, China; ogloo56@163.com (R.O.); aodeng@imnu.edu.cn (G.A.)

**Keywords:** Fenton-reaction, malignant tumor, nanoparticles

## Abstract

Cancer is a complex and multifaceted disease that continues to be a global health challenge. It exerts a tremendous burden on individuals, families, healthcare systems, and society as a whole. To mitigate the impact of cancer, concerted efforts and collaboration on a global scale are essential. This includes strengthening preventive measures, promoting early detection, and advancing effective treatment strategies. In the field of cancer treatment, researchers and clinicians are constantly seeking new approaches and technologies to improve therapeutic outcomes and minimize adverse effects. One promising avenue of investigation is the utilization of the Fenton reaction, a chemical process that involves the generation of highly reactive hydroxyl radicals (·OH) through the interaction of hydrogen peroxide (H_2_O_2_) with ferrous ions (Fe^2+^). The generated ·OH radicals possess strong oxidative properties, which can lead to the selective destruction of cancer cells. In recent years, researchers have successfully introduced the Fenton reaction into the cancer microenvironment through the application of nanotechnology, such as polymer nanoparticles and light-responsive nanoparticles. This article reviews the progress of the application of the Fenton reaction, catalyzed by polymer nanoparticles and light-responsive nanoparticles, in the cancer microenvironment, as well as the potential applications and future development directions of the Fenton reaction in the field of tumor treatment.

## 1. Introduction

Malignant tumors, also known as cancer, are one of the leading causes of death worldwide. It is a disease characterized by uncontrolled growth and proliferation of abnormal cells [1]. Malignant tumors are highly invasive and metastatic, meaning that they can invade the surrounding tissues and organs and spread to other parts of the body through the bloodstream or lymphatic system. This poses significant challenges for cancer treatment. The development of cancer is typically multifactorial, involving complex changes on multiple levels. Genetic mutations play a crucial role in the development of cancer. These mutations can be inherited or acquired through environmental factors. They disrupt normal cellular regulatory mechanisms, leading to uncontrolled cell growth and division. Such abnormal cells proliferate unchecked, forming tumors [2]. In addition to genetic mutations, several other factors are associated with cancer development. Environmental exposures are significant contributors, including high-risk factors such as smoking, excessive alcohol consumption, unhealthy dietary habits, lack of physical exercise, prolonged exposure to carcinogens and radiation, and chemical exposures in daily life. These factors can damage the cellular DNA, increasing the risk of cancer [3]. The symptoms and manifestations of cancer vary depending on the type and location of the tumor. Some common symptoms include persistent fatigue, unexplained weight loss, unexplained pain, the presence of lumps or masses, and various organ dysfunctions [4]. The development of cancer is a complex process involving multiple factors and mechanisms. Generally, cancer originates from genetic mutations or epigenetic changes in normal cells. These genetic abnormalities can arise from various factors, including naturally occurring mutations, environmental exposures, genetic predisposition, or lifestyle choices. During the development of cancer, the normal cellular regulatory mechanisms fail, leading to uncontrolled cell proliferation and growth. Cancer metastasis refers to the spread of a malignant tumor from its primary site to other parts of the body. It is a critical feature of cancer progression, and a significant challenge in treatment [5]. The process of metastasis involves multiple complex steps. Firstly, the cancer cells in the primary tumor must acquire invasiveness in order to penetrate the barriers of the surrounding tissues. They enter the bloodstream, lymphatic vessels, or body cavities, invading the circulatory system. Once in the blood or lymphatic system, the cancer cells can be transported to various organs and tissues of the body, such as the lungs, breasts, colon, prostate, liver, etc. During circulation, the cancer cells must overcome various obstacles, including immune system surveillance and clearance mechanisms by organs such as the spleen. Only a small number of cancer cells successfully leave the circulatory system and establish themselves in new tissues. Once cancer cells settle in a distant location from the primary tumor, they start proliferating and form new tumor foci. These cells can adapt and survive in the new environment, continue to invade the surrounding tissues, and eventually impact the function of the new organs or tissues. Metastasis adds complexity and challenges to cancer treatment. Once cancer cells spread to distant organs or tissues in the body, treatment becomes more difficult. This is because, at this stage, the cancer cells have formed new tumor foci in multiple sites, and they may develop resistance to certain treatment modalities [6]. Therefore, cancer remains a global, perplexing disease.

### 1.1. Tumor Microenvironment

**Figure 1:**
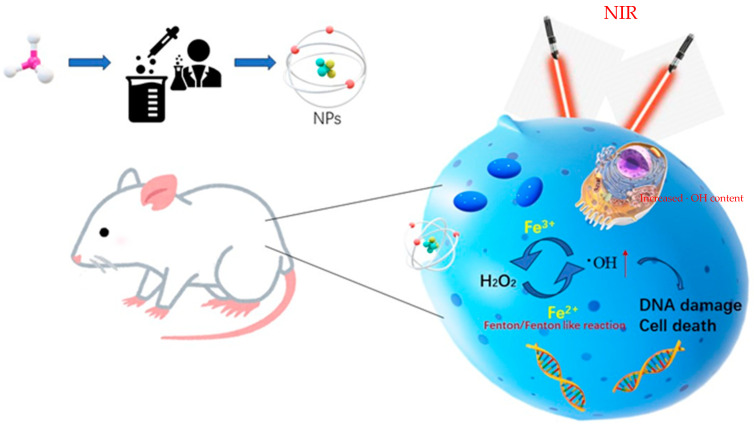
Illustration of cancer treatment using the Fenton reaction catalyzed by NPs under NIR irradiation.

The tumor microenvironment refers to the complex ecosystem formed around tumor cells. It includes various components, such as cells, blood vessels, an extracellular matrix, and immune cells surrounding the tumor cells. The tumor microenvironment is characterized by its complexity and variability, which can vary depending on individual differences and tumor types. Tumor cells exhibit a range of abnormal physiological and metabolic characteristics in the tumor microenvironment, producing large amounts of lactic acid, a condition known as acidosis [7]. Acidosis not only alters the behavior of the tumor cells, but also affects the surrounding cells and tissues. The acidic microenvironment can promote tumor cell invasion and metastasis while suppressing immune cell function, enabling tumors to evade immune surveillance. Furthermore, the tumor cells often have dysfunctional mitochondria, which can lead to an imbalance in the production and removal of reactive oxygen species, including H_2_O_2_. This oxidative stress can result in the accumulation of H_2_O_2_, contributing to the higher levels observed in tumor environments [8,9]. H_2_O_2_ is a reactive oxidizing agent that can cause oxidative damage to DNA, proteins, and lipids, and is involved in the growth, invasion, and metastasis of tumor cells. The tumor microenvironment not only provides a conducive environment and nutrients for tumor initiation and progression, but also poses challenges for selective and effective tumor treatment. The complex network structure formed by the tumor cells, surrounding cells, and extracellular matrix impedes the effective penetration of anti-tumor therapeutic agents into the tumor tissue, limiting treatment efficacy. Additionally, the presence of immune-suppressive mechanisms within the tumor microenvironment allows the tumor cells to evade immune attacks, further complicating treatment [10]. Due to the similarities between early-stage cancer symptoms and those of other diseases, cancer is often misdiagnosed or diagnosed late, presenting a significant challenge for cancer treatment and prognosis. The traditional treatment modalities for malignant tumors mainly include surgical tumor resection, chemotherapy [11], radiotherapy [12], immunotherapy, etc. The choice of the treatment regimen depends on the type and stage of the cancer, as well as the overall health status of the patient. For some early-stage diagnosed cancers, the cure rate is relatively high. However, for late-stage cancers, or cases with metastasis, the treatment becomes significantly more challenging, and the prognosis is often poor. Moreover, these treatment modalities have several limitations, such as significant side effects and the potential development of drug resistance [13]. Therefore, the search for more effective cancer treatment methods, especially in early-stage malignant tumor treatment, is crucial. In this regard, the research group led by Tang proposed a novel approach called chemodynamic therapy (CDT) in 2016 [14,15]. CDT essentially utilizes the redox reaction between ferrous ions (Fe^2+^) and H_2_O_2_ in the Fenton reaction, catalyzing the generation of highly toxic reactive oxygen species (ROS) (Figure 1). This disrupts the intracellular redox balance, and the generated free radicals can cause oxidative damage and death of DNA and other biomolecules, thereby killing the cancer cells. Therefore, the Fenton reaction and Fenton-like reactions can be considered as highly promising methods for treating malignant tumors [16]. Among them, Fenton-like reactions refer to oxidation–reduction reactions that are similar to the traditional Fenton reaction, but instead employ catalysts other than iron ions. Fenton-like reactions typically utilize other metal ions or non-metal catalysts to achieve similar oxidation–reduction processes. Transition metal ions (such as cobalt, cadmium, copper, silver, manganese, nickel, etc.) can be used in Fenton-like reactions to catalyze the generation of highly toxic ROS for tumor destruction in tumor treatment [17,18,19,20,21].

### 1.2. Fenton Reaction

The Fenton reaction is a traditional inorganic reaction named after the British chemist Henry John Horstman Fenton in the late 19th century. This reaction is characterized by its fast reaction rate, environmental friendliness, low cost, scalability, and adaptability. The Fenton reaction and its related reagents have been known for over one hundred years, and this catalytic reaction has been widely used in various applications, such as electronic waste treatment [22], wastewater treatment and water purification [23], in the pharmaceutical field [24], and environmental monitoring [25], among others. The application of the Fenton reaction in the environmental field has been extensively studied and practiced. Particularly in water treatment, the Fenton reaction can effectively remove harmful substances, such as organic matter, organic solvents, dyes, and pesticides, from water. Furthermore, the Fenton reaction can be employed in the treatment of industrial wastewater and hospital wastewater, which are complex waterbodies.

The Fenton reaction is a chemical reaction that utilizes H_2_O_2_ and ferrous ions (Fe^2+^) as reactants to generate ·OH. The reaction equations are as follows:

H_2_O_2_ + Fe^2+^ → Fe^3+^ + ·OH + OH^−^

**Figure 2:**
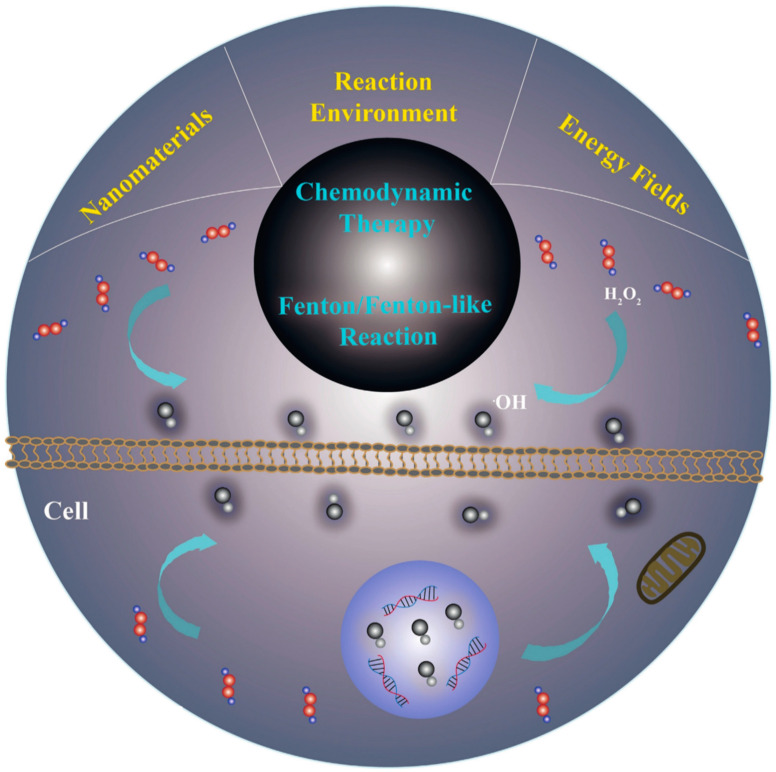
Tumor microenvironment-mediated Fenton/Fenton-like reactions [13].

H_2_O_2_ is a highly reactive oxygen species that can penetrate cell membranes and exhibit high activity and good diffusion capacity. When H_2_O_2_ enters cells at sufficient concentrations, it can induce cell death. It is believed that the functionality of the Fenton reaction in the degradation of organic pollutants is attributed to the generation of hydroxyl radicals (·OH), considering that human cells primarily consist of organic substances. Thus, it is hypothesized that the Fenton reaction could be directly introduced to destroy organic substances within cancer cells, thereby inducing cancer cell death. In the Fenton reaction, H_2_O_2_ reacts with iron ions to generate ·OH [26]. Hydroxyl radicals are one of the most important intermediate products, and they are typically considered to be the most toxic ROS generated in the Fenton reaction. These radicals possess high reactivity and can react with biological molecules such as fatty acids, proteins, and DNA, leading to oxidation and damage to these biomolecules [27]. The damage manifests as cell membrane disruption, a release of cytochrome c, and DNA strand breaks [28,29]. This tool for killing cancer cells by inflicting destructive damage on cellular components has garnered significant interest. Furthermore, the Fenton reaction can also produce hydroxide ions, which also possess certain oxidative capabilities. These ions can react similarly to hydroxyl radicals and can combine with certain elements like sodium and potassium to form unstable compounds. In the weakly acidic tumor microenvironment, cancer cells have a higher content of H_2_O_2_, making it favorable as a reactant in the Fenton reaction. Therefore, researchers have found that introducing catalysts based on the Fenton reaction and effectively triggering the Fenton reaction to generate a large quantity of toxic ·OH could be a potential method for treating malignant tumors [13] (Figure 2).

## 2. Fenton Reaction for the Treatment of Malignant Tumors

### 2.1. Polymer Nanoparticle-Catalyzed Fenton Reaction for Malignant Tumor Treatment

**Figure 3:**
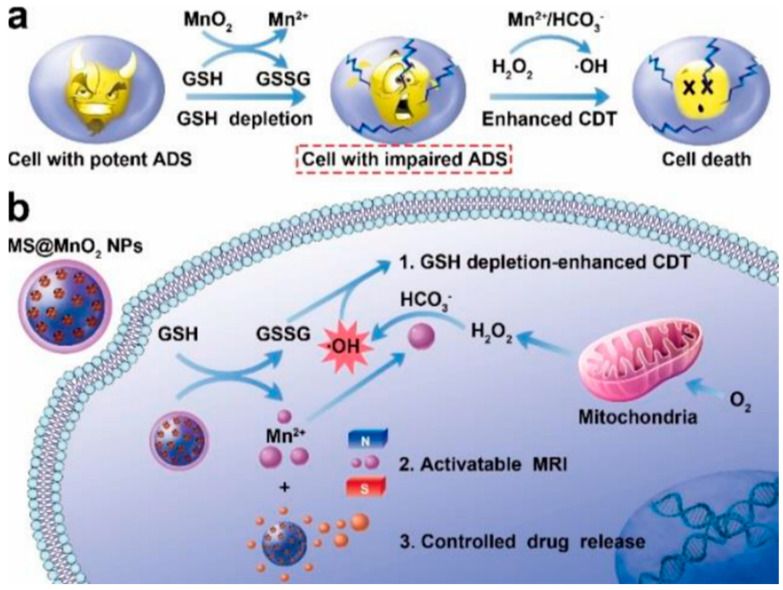
(**a**) The mechanism of MnO_2_ as an intelligent cancer cell therapeutic agent. (**b**) Schematic illustration of the application of MS@MnO_2_ nanoparticles for MRI-monitored chemodynamic combination therapy [39].

**Figure 4:**
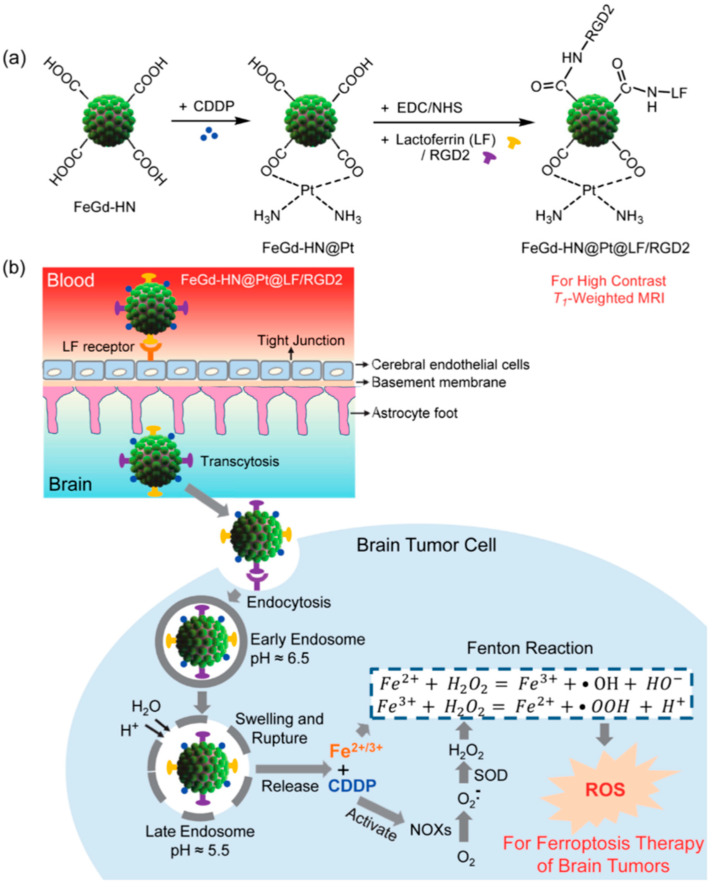
(**a**) Schematic illustration of the synthesis of FeGd-HN@Pt@LF/RGD 2. (**b**) Mechanism illustration for the ferroptosis therapy (FT) of orthotopic brain tumors with self-MRI monitoring [40].

**Figure 5:**
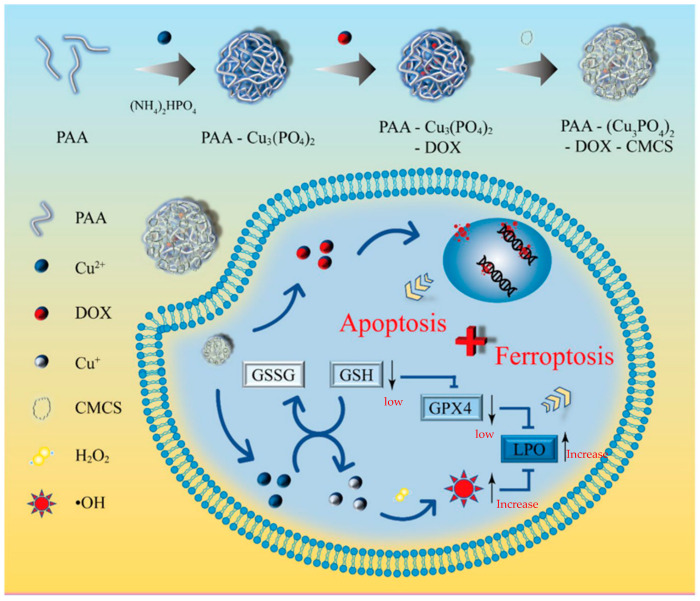
The preparation process and therapeutic mechanism of PAA-Cu3(PO4)2-DOX-CMCS NPs [46].

**Figure 6:**
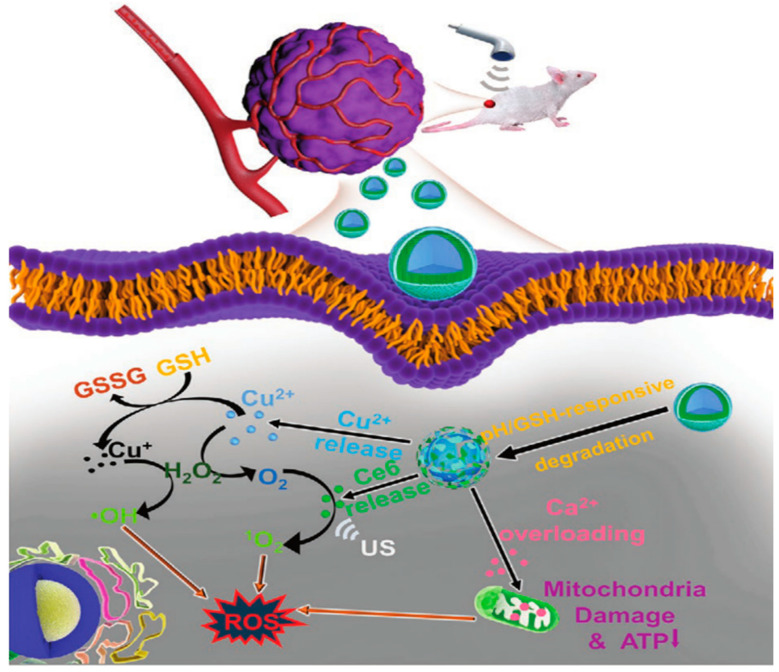
Schematic illustration of the reaction mechanism of Cu/CaCO3@Ce6 NPs [49].

**Figure 7:**
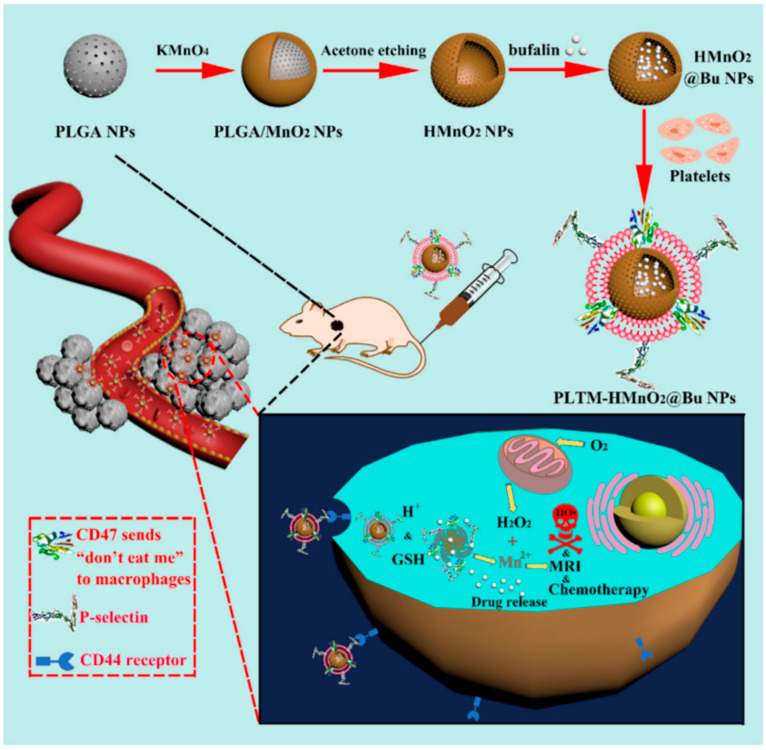
Schematic illustration of the synthesis and therapeutic mechanism of PLTM-HMnO_2_@Bu NPs [71].

**Figure 8:**
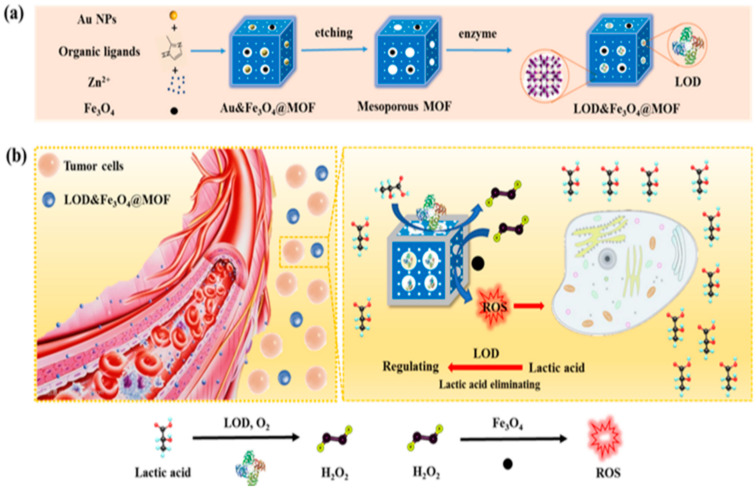
(**a**) Illustration of the synthesis of LFZ NPs. (**b**) Tandem biological-chemical catalytic reactions for effective catalytic tumor treatment based on the characteristic of the TME [56].

**Figure 9:**
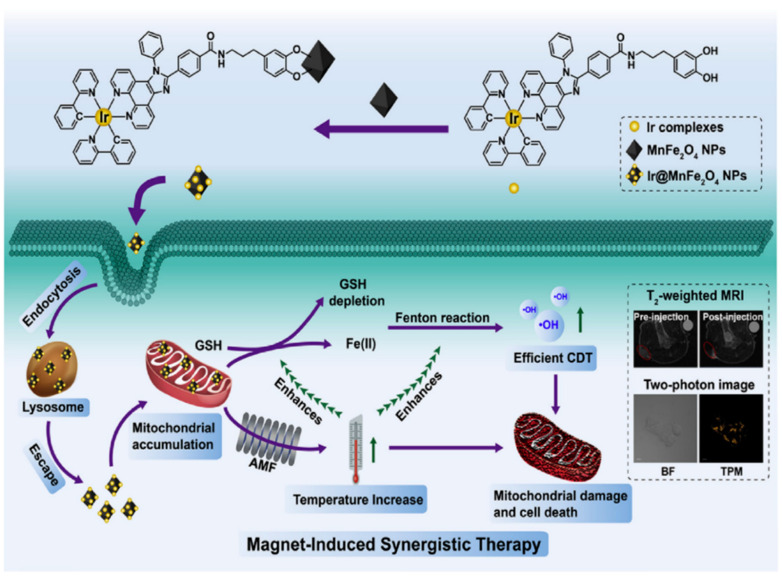
Schematic illustration of the synthesis and therapeutic mechanism of Ir@MnFe_2_O_4_ NPs [74].

In recent years, with the continuous advancement of technology, nanotechnology has made significant progress in various fields of application in China. Nanotechnology has found widespread application in traditional materials, biochemistry, medicine, and other fields. Compared to the past, the preparation methods of nanomaterials have made great strides, becoming more scientific and simplified [30]. Nowadays, nanomaterials have garnered increasing attention from researchers, as they exhibit their charm in various domains [31]. Due to the low efficiency of the Fenton reaction in malignant tumors, and the limited generation of ·OH, it is difficult to effectively eliminate cancer cells. To address this issue, researchers have devised numerous methods to catalyze the Fenton reaction and improve its therapeutic efficacy against malignant tumors [32]. Among these approaches, the use of nanocomposite materials for catalyzing the Fenton reaction in the treatment of malignant tumors has gained attention. The research group led by Sun utilized a simple oxidative self-assembly strategy, employing Fe(II) and noble metal salts to construct gold@FexOy core-shell nanoparticles in a one-step process [33]. This included Au@FexOy NPs, AuRu@FexOy NPs, AuPt@FexOy NPs, and AuPd@FexOy NPs. Compared to conventional AuPd@c-Fe_2_O_3_ nanocrystals, the AuPd@FexOy nanoparticles with a metastable FexOy shell exhibited significantly enhanced generation of hydroxyl radicals (·OH) in the Fenton reaction activated by a small amount of NaBH_4_. This research contributes to the rational design of nanomedicines with controllable diagnostic and therapeutic properties within the body. The research group led by Wu developed Fe(III)@WS_2_-PVP nanoparticles with enhanced biodegradability and drug loading capacity. Within these nanoparticles, an oxidation–reduction reaction occurs between Fe(III) species and WS_2_, resulting in the continuous generation of Fe^2+^ and WO_4_^2−^. This intrinsic redox reaction leads to the enhanced biodegradation of the nanoparticles and drug release. Furthermore, the higher levels of H_2_O_2_ and the acidic conditions in the tumor microenvironment accelerate the generation of Fe^2+^ and drug release, further promoting the Fenton reaction within tumor cells, and generating a large amount of highly toxic ·OH for nanocatalytic tumor therapy. This concept provides an innovative approach and new avenues for the design of efficient cancer treatments using degradable theranostic agents [34]. The research group led by Koo developed a heterogeneous chemodynamic therapy system specifically targeting tumors, utilizing copper–iron peroxide nanoparticles (CFp NPs) that respond to the tumor microenvironment for synergistic therapy. CFp NPs undergo decomposition under slightly acidic conditions, self-supplying H_2_O_2_, and releasing Cu and Fe ions, which collaboratively generate hydroxyl radicals through efficient catalytic cycling, thereby exhibiting excellent therapeutic efficacy in tumors [35]. Unlike independent nanoparticles, the Cu^+^-assisted conversion of Fe^3+^ to Fe^2+^ in this system enables synergistic effects that can achieve nearly complete tumor eradication with minimal therapeutic dosages, without the need for additional treatment modalities [36]. These findings demonstrate the tremendous potential of these nanoparticles in cancer therapy, providing another promising approach for the treatment of malignant tumors. The research group led by Zheng recently conducted a study on targeted tumor therapy using novel nanoparticles called HMON-GOx@MnO_2_ NPs as a treatment strategy [37]. These nanoparticles possess stimulus-responsive properties, with an outer layer composed of MnO_2_ and a core containing glucose oxidase (GOx)-loaded nanomaterial. The HMON-GOx@MnO_2_ NPs formed by the encapsulation of the enzyme and manganese dioxide shell enable dual-modal molecular imaging-guided tumor radiosensitization. When the HMON-GOx@MnO_2_ NPs enter the tumor microenvironment, the MnO_2_ shell reacts with intracellular reduced glutathione (GSH), releasing Mn^2+^ and triggering an endogenous Fenton-like reaction with H_2_O_2_, leading to the generation of hydroxyl radicals (·OH) and promoting iron-dependent cell death. Furthermore, GOx catalyzes the oxidation of glucose to produce H_2_O_2_, further enhancing the iron-dependent cell death induction by the HMON-GOx@MnO_2_ NPs. Since the balance of H_2_O_2_ and GSH in tumor tissues changes dynamically, to minimize interference from gastric acid secretion and enhance the induction of iron-dependent cell death, the researchers introduced a proton pump inhibitor (PPI) that can reduce gastric acid secretion and decrease the degradation of glucose oxidase by gastric acid, thereby further enhancing the self-generation of H_2_O_2_ and improving the induction of iron-dependent cell death. The results showed that the addition of the PPI significantly improved the induction of iron-dependent cell death, in addition to using the HMON-GOx@MnO_2_ NPs alone. Additionally, the researchers utilized HMON-GOx@MnO_2_ NPs as fluorescent probes for photoacoustic imaging (PA) and responsive T1-weighted magnetic resonance imaging (MRI) to monitor their distribution in the tumor region [38]. This nanocatalyst exhibited an excellent anti-tumor performance and radiosensitization effects in the tumor microenvironment, making it suitable for tumor treatment guided by dual-modal molecular imaging. This strategy provides a new approach for precise, effective, and safe tumor therapy by combining nanomaterials with drugs, utilizing stimulus-responsive mechanisms and molecular imaging technologies. Nanocatalysts can release hydroxyl radicals through a mechanism similar to the Fenton reaction, thereby killing cancer cells. Compared to traditional Fenton reactions, these nanoparticles have more complex designs and functionalities [39] (Figure 3). The research group led by Shen synthesized Fe_3_O_4_/Gd_2_O_3_ hybrid nanoparticles loaded with cisplatin (CDDP), combined with lactoferrin (LF) and RGD peptide dimer (RGD2), referred to as FeGd-HN@Pt@LF/RGD2 nanoparticles [40]. These nanoparticles can penetrate the blood–brain barrier and enter tumor cells through LF receptor-mediated transport. Inside of the cells, during the endocytosis and degradation process, Fe^2+^, Fe^3+^, and CDDP can be released from the nanoparticles, which are direct reactants involved in the Fenton reaction. Additionally, the released CDDP can activate NADPH oxidases (NOXs), producing H_2_O_2_, further accelerating the Fenton reaction. Thus, the reactants (Fe^2+^, Fe^3+^, and H_2_O_2_) involved in the Fenton reaction can be successfully delivered to the tumor site, accelerating the reaction, and generating reactive oxygen species (ROS) to induce cancer cell death. In in vivo experiments, the researchers found that FeGd-HN@Pt@LF/RGD2 nanoparticles successfully suppressed tumor growth and prolonged the survival time of mice in an orthotopic brain tumor model, with higher nanoparticle uptake being observed, compared to the control group. The results have demonstrated that the FeGd-HN@Pt@LF/RGD2 nanoparticles successfully delivered the reactants involved in the Fenton reaction to the tumor site and effectively inhibited tumor growth [40] (Figure 4). This nanoparticle can provide iron cell death therapy efficacy for in situ brain tumors. The research group led by Xu developed a multifunctional nanoplatform using Mn (0.25)-Fe_3_O_4_-III nanoparticles for tumor therapy and diagnosis. By controlling the amount of water, they successfully synthesized nanoparticles with tunable porous structures [41]. The Mn (0.25)-Fe_3_O_4_-III nanoparticles exhibit superior Fenton reaction activity compared to other iron-based nanoparticles, providing significant advantages for iron-induced cancer cell death therapy. The researchers loaded the anticancer drug doxorubicin (DOX) onto the nanoparticles, enabling controlled drug release [42]. In the tumor microenvironment, this nanoplatform demonstrates multiple anticancer functions, as the nanoparticles can generate reactive oxygen species through a catalytic Fenton reaction and release the chemotherapy drug DOX in order to induce tumor cell death. In vitro and in vivo experiments have demonstrated that DOX/Mn (0.25)-Fe_3_O_4_-III nanoparticles exhibit efficient drug delivery, precise targeted activation, reduced off-target toxicity, significant synergistic anticancer effects, and excellent magnetic resonance imaging performance [41]. Dox–metal complexes are a potential new anticancer agent with a higher efficacy than free DOX [43]. This multifunctional nanoplatform holds promising clinical application prospects, providing a new strategy and approach for integrated diagnosis and treatment. Copper-based nanomaterials have shown great potential in tumor therapy, such as copper sulfide nanoparticles and copper phosphate (Cu_3_(PO_4_)_2_) nanoparticles for tumor therapy [44,45]. The research group led by Zhao synthesized polyacrylic acid-phosphocopper-doxorubicin-carboxymethyl chitosan (PCPDC) nanoparticles. These nanoparticles are degraded under the acidic conditions of tumor cells, resulting in the generation of Cu^2+^ ions. The Cu^2+^ ions react with glutathione (GSH) to form Cu^+^, while depleting GSH. H_2_O_2_ is converted to hydroxyl radicals through a Cu^+^-mediated Fenton-like reaction. The depleted GSH downregulates the expression of glutathione peroxidase 4 (GPX4) protein, leading to the accumulation of lipid peroxides and enhancing ferroptosis. The released doxorubicin effectively induces apoptosis of tumor cells [46] (Figure 5). These nanoparticles can induce simultaneous apoptosis and ferroptosis in tumor cells through multiple mechanisms, demonstrating a great therapeutic potential. The research group led by Ling synthesized pH-responsive CPNS@Pt nanoparticles [47]. These nanoparticles can decompose into Cu^2+^ and H_2_O_2_ in the tumor microenvironment and generate hydroxyl radicals (·OH) through a Fenton-like reaction process. Simultaneously, Pt nanoparticles are also released, exhibiting enzymatic activities similar to oxidases and peroxidases, catalyzing a series of cascade oxidation reactions and inducing oxidative damage within cells [48]. These nanoparticles not only alleviate the hypoxic environment of tumors, but also efficiently generate reactive oxygen species (ROS) without the need for external stimuli, due to their dependence on an acidic pH. Compared to traditional ROS generation systems, this nanocomposite material offers advantages such as easy synthesis, simple operation, and low toxicity of nanomaterials. These features endow it with a tremendous potential for cancer treatment. The research group led by Zhao synthesized a novel Cu-doped CaCO_3_ nanoparticle (Cu/CaCO_3_@Ce_6_, CCC NPs) [49]. It was found that the presence of glutathione (GSH) in tumor cells accelerates the degradation of CCC NPs, ensuring the release rate of functional Cu and Ca ions. As a Fenton-like agent, CCC NPs can be activated in the tumor microenvironment (TME) by overexpressed H_2_O_2_, generating hydroxyl radicals (·OH). Furthermore, through the reduction in Cu^2+^, excessive intracellular GSH is depleted, providing Cu^+^ for the Fenton reaction and promoting ·OH generation (Figure 6). This study emphasizes a TME-sensitive strategy, opening up promising avenues for ROS-based cancer therapy. The research group led by Cong developed a Ru-based nanozyme with dual catalytic activities (glucose-oxidase-like activity and peroxidase-like activity). The nanozyme was loaded into nanoliposomes and modified with a tumor cell membrane to obtain Ru@ATO-Lip/M nanoparticles [50,51]. Ru exhibits GOx-like activity, strongly catalyzing the reaction between glucose and oxygen to generate H_2_O_2_. Under the acidic conditions of the tumor microenvironment, Ru catalyzes the generation of ·OH through a Fenton-like reaction with H_2_O_2_. Due to the tumor cell membrane modification on the surface of the nanozyme, Ru@ATO-Lip/M nanoparticles are more easily taken up by tumor cells, enabling targeted therapy. Additionally, ATO, as an oxygen-consuming competitive inhibitor, can improve the hypoxic conditions of the tumor microenvironment and provide sufficient oxygen for the GOx-like activity of Ru [52]. The sequential catalytic action of this nanozyme achieves sustained starvation therapy and a Fenton-like reaction, resulting in sustained synergistic therapeutic effects. This research provides a foundation for the further exploration of nanozyme activity and anti-tumor applications, opening up new research directions for nanocatalytic drugs [53]. The research group led by Li synthesized a multifunctional nanoparticle based on hollow Prussian blue (HPB), called HPB-CHC/LOD@PEG (referred to as HCLP NPs) [54]. HCLP NPs are designed to interfere with lactate metabolism in the tumor microenvironment, exhibiting the ability to inhibit tumor growth and metastasis. The nanoparticles utilize hollow Prussian blue as a carrier and load α-cyano-4-hydroxycinna-mate (CHC) and lactate oxidase (LOD) within it, with polyethylene glycol (PEG) being used for encapsulation [55]. In the tumor microenvironment, HCLP NPs release CHC and LOD. CHC inhibits lactate uptake and reduces lactate aerobic respiration, while LOD catalyzes the breakdown of lactate, generating hydrogen peroxide (H_2_O_2_). The released H_2_O_2_ can participate in Fenton-like reactions, interacting with the metal ions within the carrier to produce highly toxic hydroxyl radicals (·OH) [55,56,57]. By inhibiting lactate metabolism and generating toxic reactants, HCLP NPs can suppress tumor growth and metastasis while enhancing therapeutic efficacy. The research group led by Liu synthesized pillar[6]-arene-based nanoparticles, known as GOx@T-NPs [58,59]. These nanoparticles are associated with the Fenton reaction and have been applied for the treatment of malignant tumors [60]. GOx@T-NPs can catalyze the conversion of glucose into hydroxyl radicals (·OH), resulting in the generation of high levels of ·OH through the Fenton reaction. This enables efficient tumor therapy. GOx@T-NPs release the chemotherapeutic drug glutathione-induced camptothecin (CPT) and selectively release it in the presence of glutathione (GSH). This enhances the sensitivity of tumor cells to chemotherapy and reverses drug resistance [61]. By releasing CPT and depleting GSH, the oxidative stress level is increased, further enhancing the production of ·OH. This combined effect achieves the synergistic effect of starvation therapy and chemotherapy [62]. Pillar[6]-arene-based nanoparticles, such as GOx@T-NPs, serve as intelligent nano-systems, capable of achieving efficient, targeted, and multimodal synergistic therapy in the treatment of malignant tumors, offering significant potential for clinical applications [63]. The research group led by Chen developed a nanoparticle system called PC-Mn@Dox-NPs, where doxorubicin (Dox) and manganese ions (Mn^2+^) are co-encapsulated within a regenerated silk fibroin matrix and surface-modified with phycocyanin (PC) [64,65]. This nanoparticle system can release Dox and Mn^2+^ in response to various stimuli, such as an acidic pH, hydrogen peroxide (H_2_O_2_), and glutathione. The released Dox inhibits the growth of tumor cells and generates a large amount of H_2_O_2_. Simultaneously, the released Mn^2+^ converts H_2_O_2_ into cytotoxic hydroxyl radicals (·OH) and oxygen (O_2_) through a Fenton-like reaction. The generated oxygen can also participate in other therapeutic processes. This nanoparticle system exhibits excellent anticancer efficacy and demonstrates good biocompatibility, offering a promising strategy for combination cancer therapy [66]. The research group led by Wang synthesized a carrier-free nanoparticle system called Mn-ZnO_2_, which achieves synergistic anticancer therapy by releasing dual ions (Mn^2+^ and Zn^2+^) and reactive oxygen species (ROS) to simultaneously regulate the degradation and activation of the p53 protein at the tumor site [67]. The nanoparticles rapidly release H_2_O_2_ and Zn^2+^ in the acidic microenvironment of cells, enhancing endogenous ROS generation and inducing the degradation of the mutant p53 protein (Mutp53) and the production of toxic hydroxyl radicals (·OH) through a Fenton-like reaction. This carrier-free nanoparticle provides a simple and efficient approach to the design of multifunctional nano systems [67]. The research group led by Cheng developed a mitochondria-targeting INH-supported WSSe/MnO_2_ nanocomposite, which can induce chemical reactions and photothermal effects within tumor cells, leading to the generation of highly toxic ·OH for tumor treatment [68]. Building upon this, the Chen research group synthesized Fe_3_O_4_@MnO_2_ nanoparticles, which consist of a core-shell structure with an iron oxide (Fe_3_O_4_) core and a manganese dioxide (MnO_2_) shell. In malignant tumors, these nanoparticles can catalyze the Fenton reaction to decompose hydrogen peroxide (H_2_O_2_) into hydroxyl radicals (·OH), thereby enhancing the efficacy of radiotherapy. Additionally, the nanoparticles achieve tumor-targeted therapy under the guidance of a magnetic field [69]. The research group led by Zhang prepared a multifunctional hollow mesoporous manganese dioxide-based (H-MnO_2_) nanoplatform, known as H-MnO2@AFIPB@PDA@Ru-NO@FA (referred to as MAPRF NPs) [70]. MAPRF NPs possess characteristics that respond to the tumor microenvironment within cancer cells, enabling them to disrupt the antioxidant defense system by consuming glutathione (GSH) and facilitating on-demand drug release. The released Mn^2+^ undergoes a Fenton-like reaction with endogenous H_2_O_2_, generating highly toxic hydroxyl radicals (·OH). This nanoplatform provides new insights for the development of novel nanoplatforms for safe, efficient, and precise cancer treatment [70]. The research group led by Wang synthesized platelet membrane-functionalized bufalin-loaded HMnO_2_ nanoparticles (PLTM-HMnO_2_@Bu NPs) for active targeting and tumor microenvironment-responsive MRI-guided chemodynamic therapy [71]. These nanoparticles possess a hollow structure that enables high bufalin loading and the responsive release of bufalin and Mn^2+^ in simulated tumor environments. Mn^2+^ not only exhibits a strong contrast enhancement in T1-weighted MRI, but also generates toxic hydroxyl radicals (·OH) through a Fenton-like reaction, leading to drug degradation. In vitro experiments have demonstrated the good biocompatibility of these nanoparticles with L929 cells, and in vivo experiments have shown effective cytotoxicity against cancer cells, particularly with improved outcomes in combined chemodynamic therapy after bufalin loading. In vivo MRI and immunofluorescent staining have revealed the accumulation of nanoparticles at the tumor site, demonstrating active targeting capabilities (Figure 7). These findings suggest that platelet-membrane-functionalized HMnO_2_ nanoparticles are a promising drug carrier for anticancer therapy, enabling MRI monitoring and the enhanced targeted treatment of tumors. This nanoparticle system holds potential for precise targeted release and enhanced diagnostics in cancer treatment. The research group led by Chu conducted studies on cancer treatments using copper-cysteamine nanoparticles (Cu-Cy NPs) for a heterogeneous Fenton-like reaction [72,73]. In the well-known Fenton reaction, hydrogen peroxide (H_2_O_2_) reacts with copper ions (Cu^2+^) under appropriate conditions to generate highly reactive hydroxyl radicals (·OH), which possess strong oxidative properties and can destroy cancer cells. In Cu-Cy nanoparticles, copper is in a reduced state (Cu^+^), exhibiting catalytic activity similar to the Fenton reaction. It can react with hydrogen peroxide (H_2_O_2_) in the tumor microenvironment to generate hydroxyl radicals (·OH), thereby selectively inducing cytotoxicity in the cancer cells. Therefore, Cu-Cy nanoparticles utilize the principle of the Fenton reaction to selectively kill cancer cells by producing highly active hydroxyl radicals. The catalytic effect of Cu-Cy nanoparticles is significantly enhanced under slightly acidic conditions, further improving their selective cytotoxicity towards cancer cells, as tumor cells exhibit acidic characteristics [72]. Cu-Cy nanoparticles demonstrate excellent stability and low toxicity towards cancer cells, providing potential opportunities for the development of translationally viable nanomedicines with low systemic toxicity in cancer therapy. This study demonstrates the novel approach of combining a heterogeneous Fenton-like reaction with nanoparticles for cancer treatment, offering a promising strategy for achieving a highly selective cancer therapy and potentially playing a significant role in clinical translation [73]. The research group led by Zhou synthesized a nanocarrier called LOD and Fe_3_O_4_@ZIF-8 nanoparticles (LFZ NPs) by loading lactate oxidase (LOD) and Fe_3_O_4_ nanoparticles into a ketimine acid salt framework-8 (ZIF-8) (Figure 8). In TEM, the LOD in the nanoparticles converts lactate into hydrogen peroxide (H_2_O_2_), which can be converted into highly toxic hydroxyl radicals (·OH) through the Fenton-like reaction catalyzed by Fe_3_O_4_ nanoparticles in the tumor region. This ultimately leads to the death of the tumor cells. Additionally, the LOD in LFZ NPs regulates the tumor microenvironment (TME) and cell cycle by consuming lactate, inducing tumor cell apoptosis. The construction of the cascade reaction was verified through in vitro cell-killing experiments. LFZ NPs exhibited effective synergistic effects in 4T1-tumor-bearing mice. Immunohistochemistry experiments have demonstrated that LFZ NPs could inhibit tumor tissue proliferation and induce apoptosis [56]. This system enables dual-mode therapy based on lactate and serves as an effective nanocatalyst for tumor suppression, providing a simple, efficient, and safe approach for cancer treatment. The research group led by Shen synthesized magnetic-temperature-responsive nanoenzyme Ir@MnFe_2_O_4_ NPs by the surface modification of lipophilic iridium (III) complex (Ir) onto MnFe_2_O_4_ NPs, which targeted mitochondria (Figure 9). These nanoparticles exhibited excellent magnetic-temperature properties and could non-invasively and locally treat cancer. Under exposure to an alternating magnetic field, Ir@MnFe_2_O_4_ NPs induced localized hyperthermia, resulting in mitochondrial damage and cell death. Furthermore, the overexpression of glutathione (GSH) in mitochondria reduced iron (III) on the nanoparticle surface to iron (II), triggering the Fenton-like reaction to generate hydroxyl radicals (·OH). The increase in iron (II) catalyzed the production rate of ·OH, further enhancing the therapeutic effect. Moreover, due to their outstanding magnetic and optical properties, Ir@MnFe_2_O_4_ NPs could be used for in vitro two-photon microscopy imaging and in vivo magnetic resonance imaging, enabling more precise and efficient cancer treatment. In summary, Ir@MnFe_2_O_4_ NPs integrate chemical dynamic therapy, magnetic-temperature therapy, two-photon microscopy imaging, and magnetic resonance imaging into a single nanoplatform, offering an efficient and precise approach for cancer treatment [74].

In summary, the approach of using polymer nanoparticles to catalyze Fenton-like reactions for the treatment of malignant tumors offers advantages such as high efficacy, targeted therapy, and biocompatibility. By generating hydroxyl radicals, it induces oxidative stress in tumor cells and achieves therapeutic effects. However, this method faces technical challenges and issues regarding biostability. It requires addressing the consistency and stability of particle synthesis while ensuring safety for human application. Further experimental and clinical trials are needed in order to evaluate its clinical prospects.

### 2.2. Application of Photothermal-Enhanced Fenton-like Reaction Using Nanoparticles in the Tumor Microenvironment

In current research, combination therapy has been widely utilized to achieve more effective tumor suppression [75]. The synergistic effect of combination therapy minimizes the harm to the human body during the treatment process [76,77]. Multifunctional nanomaterials have been extensively studied for photothermal therapy (PTT), which involves the utilization of materials capable of absorbing near-infrared (NIR) light and efficiently converting it into heat while exhibiting excellent biocompatibility [78]. PTT is a therapeutic strategy for treating malignant tumors by inducing hyperthermia using NIR radiation, which delivers photothermal agents to the tumor site to suppress and eliminate the cancer cells [79,80,81]. PTT offers advantages such as high selectivity, minimal invasiveness, low toxicity, deep tissue penetration, high spatial resolution, and multifunctionality, bringing new treatment approaches and prospects to the field of cancer therapy [81,82,83]. The combination of PTT and Fenton-like reactions can achieve synergistic therapeutic effects by harnessing the unique properties of nanomaterials. This combined approach exerts a dual attack on tumor cells through localized heating via photothermal effects and the generation of oxygen radicals through Fenton-like reactions, thereby enhancing the treatment efficacy and reducing side effects. Consequently, the strategy of utilizing photothermal nanoparticles to catalyze Fenton-like reactions has gained significant attention in recent years [84,85].

**Figure 10:**
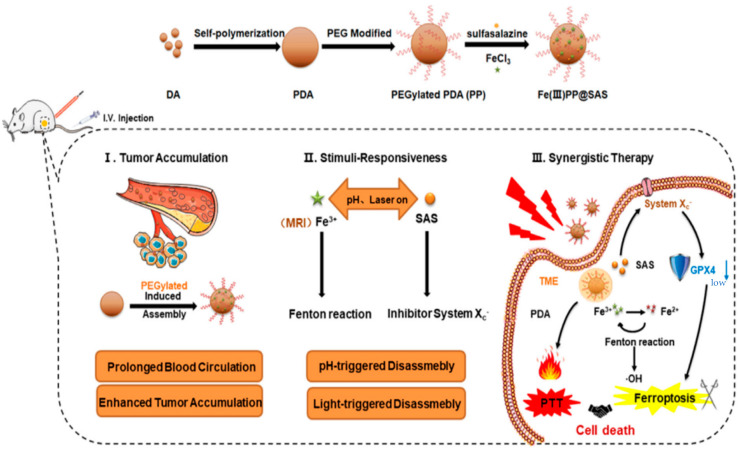
Schematic illustration of fabrication of the pro-ferroptotic Fe(III)PP@SAS NPs and their therapeutic mechanism [88].

**Figure 11:**
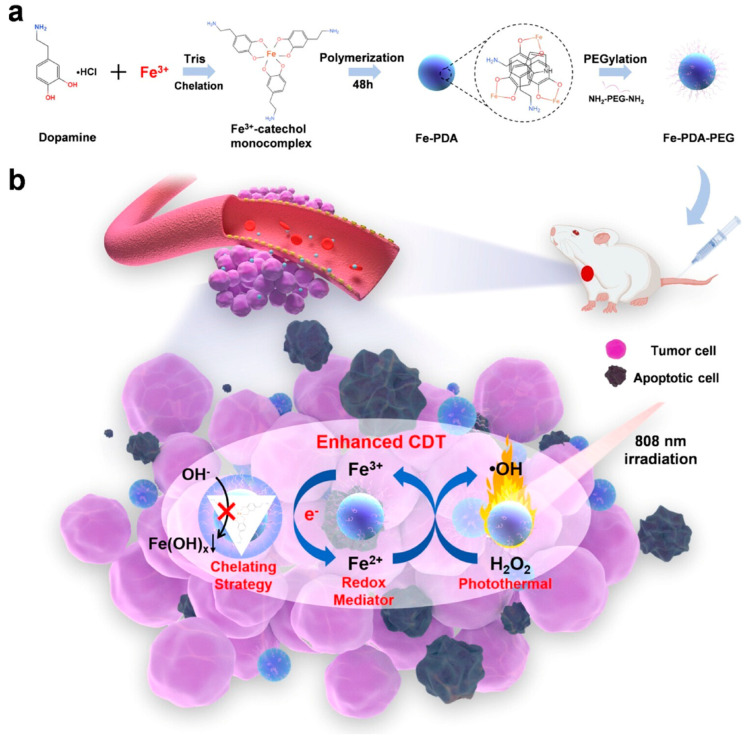
(**a**) Schematic illustration of the synthesis procedure. (**b**) Mechanism for Fe-PDA-PEGNP-mediated pH-independent photothermal-chemodynamic tumor therapy [93].

**Figure 12:**
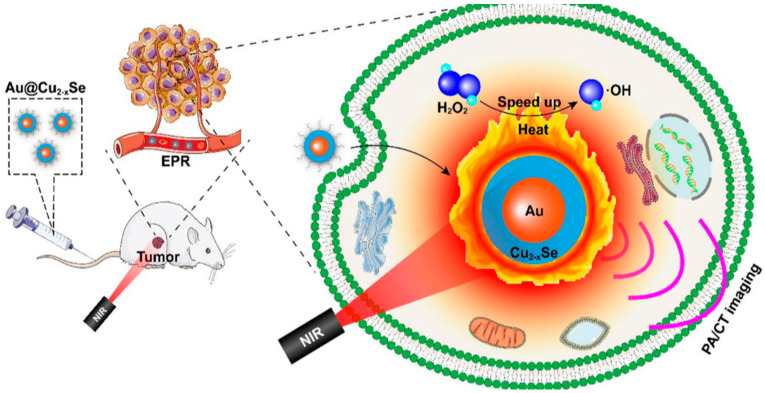
Schematic illustration of core-shell Au@Cu2-xSe NPs for PA/CT imaging-guided PTT synergistic cancer therapy [101].

**Figure 13:**
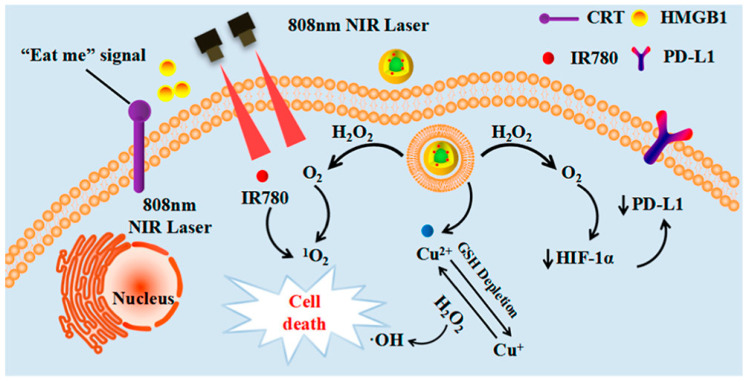
Mechanism of ZHTC@IR780 NPs inducing tumor cell apoptosis [104].

**Figure 14:**
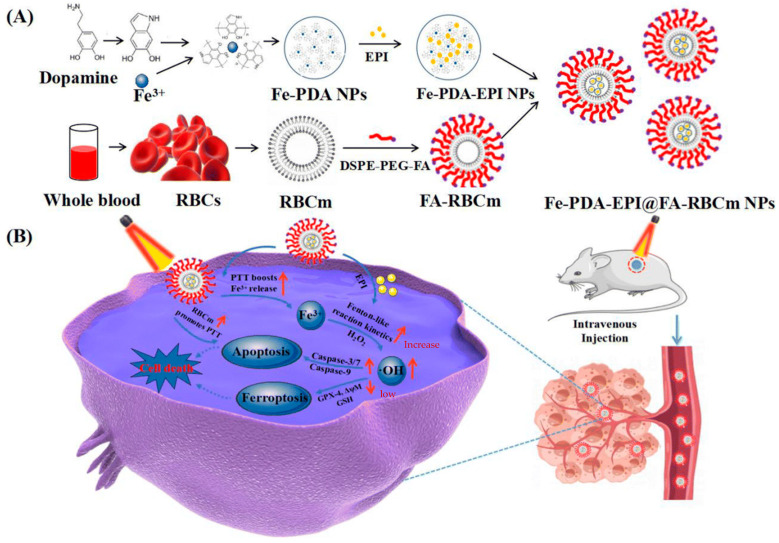
(**A**) Preparation of Fe-PDA-EPI@FA-RBCm NPs. (**B**) Schematic illustration of NIR-mediated PTT and Fenton-like mechanisms that induce synergistic ferroptosis-PTT [111].

**Figure 15:**
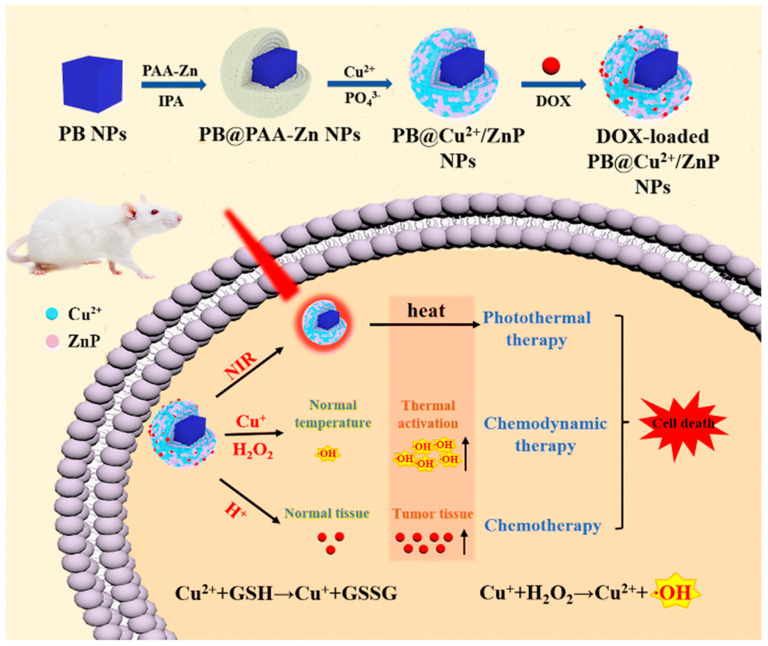
Schematic illustration of the synthesis and therapeutic mechanism of PB@Cu^2+^/ZnP NPs [113].

The research group led by Cheng developed a combination therapy platform based on bioinspired metal–organic frameworks (MOFs), named PPy-CTD@MIL-100@MPCM nanoparticles (PCMM NPs) [86,87]. Under NIR irradiation, PCMM NPs can precisely target tumor tissues through active targeting with minimal adverse effects on normal tissues. They can rapidly heat up and trigger drug release. In the tumor microenvironment, the heat generated by hyperthermia can accelerate the release of iron ions from the PCMM NPs, thereby enhancing the efficiency of the Fenton reaction and generating highly reactive ·OH. These radicals can disrupt the biomolecules in the tumor cells, leading to cell death and apoptosis [88]. Therefore, PCMM NPs offer a new strategy for tumor treatment by achieving efficient Fenton reactions. The research group led by Zhu synthesized multifunctional nanoparticles, Fe(III)PP@SAS NPs, by loading iron ions and sunitinib [88] (Figure 10). These nanoparticles enable dual iron death therapy. The iron ions in the nanoparticles generate ·OH through the Fenton reaction, acting as “swords” to attack cancer cells. Additionally, sunitinib inhibits the xCT/GPX4 axis, disrupting the cancer cells’ defense against iron death [89]. Furthermore, NIR irradiation and acidic stimulation can accelerate the release of the loaded substances, further enhancing the efficacy of the iron death therapy [90]. Iron ions can also be used as magnetic resonance imaging agents for tumor visualization and treatment assessment. The research group led by Zhao synthesized polyethylene glycol (PEG)-modified iron-polydopamine (Fe-PDA-PEG) nanoparticles (Fe-PDA-PEG NPs) [91,92]. These nanoparticles are capable of the efficient generation of hydroxyl radicals (·OH) through the Fenton reaction at a neutral pH. Additionally, the nanoparticles exhibit remarkable photothermal performance. Fe-PDA-PEG NPs can achieve excellent photothermal therapy effects under near-infrared (NIR) irradiation, promoting the generation of ·OH in the tumor microenvironment [93] (Figure 11). Overall, this study opens up new avenues for enhancing the anti-tumor activity of nanomedicines in weakly acidic, or even neutral, microenvironments. The research group led by Zhang synthesized core-shell-structured Au@Cu^2^-xSe nanoparticles [94]. The Au@Cu^2^-xSe NPs achieve efficient photothermal conversion through the localized surface plasmon resonance (LSPR) coupling effect in the core-shell structure [95,96]. The generated heat can accelerate Fenton-like reactions on the Cu^+^ sites, leading to the production of a large amount of hydroxyl radicals (·OH) within the tumor cells, causing irreversible damage and inhibiting tumor growth. These nanoparticles not only achieve the therapeutic effect of photothermal therapy (PTT), but also utilize the heat generated during the photothermal process to enhance the efficacy of chemodynamic therapy (CDT), thereby achieving a combined PTT + CDT therapeutic effect and suppressing tumor growth. The research group led by Qiu synthesized polyethylene glycol (PEG)-modified copper-(II)-chelated polydopamine (PDA-Cu (II)-PEG) nanoparticles. After NIR irradiation, the nanoparticles generate heat and increase the reaction temperature. The copper ions (Cu (II)) within the nanoparticles are released at high temperatures and react with H_2_O_2_ to form a Fenton-like reaction, generating hydroxyl radicals (·OH) that induce cell apoptosis and damage. Therefore, the photothermal-enhanced nanoparticles effectively catalyze the Fenton reaction and generate ·OH radicals to combat the tumor cells [97]. The research group led by Wang developed intelligent multifunctional biomimetic nanoparticles (CS-I/J@CM NPs) composed of ultra-small Cu2-xSe nanoparticles for the treatment of glioblastoma (GBM) [98,99]. Under NIR irradiation, these nanoparticles generate a large amount of reactive oxygen species (ROS) and oxygen [100]. The ROS and oxygen react with H_2_O_2_ in the tumor to produce highly cytotoxic ·OH through a Fenton-like reaction. The resulting ·OH from the Fenton-like reaction exhibits strong cytotoxicity against the tumor cells and induces immunogenic cell death (ICD), thereby activating anti-tumor immune responses. Additionally, the Fenton-like reaction can regulate the immunosuppressive microenvironment of GBM, promote tumor cell death, enhance immune responses, and improve the efficacy of immunotherapy for GBM [101] (Figure 12). The research group led by Su prepared polydopamine nanoparticles (PDA NPs) and nanoparticles composed of epigallocatechin gallate (EGCG) and an Fe-metal–polyphenol network (EGCG@PDA) [102]. Under NIR irradiation, the PDA nanoparticles generate heat, creating a localized high-temperature environment. This high-temperature environment promotes the release of EGCG and Fe^3+^ from the metal–polyphenol network. EGCG can reduce Fe^3+^ to Fe^2+^ and react with the excessive H_2_O_2_ present in cancer cells, generating highly oxidative hydroxyl radicals of ·OH [103]. Compared to other typical PDA-based PTT/CDT combination therapy systems, the preparation of EGCG@PDA is simpler, and the introduction of EGCG/Fe MPN also yields good therapeutic outcomes. The research group led by Li synthesized multifunctional nanoparticles based on zein hydrolysates (ZHTC-HA-TA-Cu@IR780) [104]. When the nanoparticles are exposed to near-infrared light, IR780 generates oxygen (O_2_) for photodynamic therapy. The Cu^+/2+^ in the nanoparticles undergoes a Fenton-like reaction in the presence of H_2_O_2_, catalyzing the decomposition of H_2_O_2_ and producing ·OH. Through this Fenton-like catalytic effect, the nanoparticles can further enhance the cytotoxic effect on tumor cells. This work provides an innovative strategy for a synergistic and comprehensive cancer therapy and suggests zein hydrolysates as a promising candidate for cancer treatment nanoparticles, which have significant potential [104] (Figure 13). The research group led by Yang combined gallic-acid-functionalized iron nanoparticles (GA-Fe NPs) and the chemotherapeutic drug cisplatin (CDDP) to form Pt-GA-Fe NPs [105]. Under NIR irradiation, CDDP activates NADPH oxidase (NOXs) to generate O^2−^, thereby increasing the level of H_2_O_2_. The H_2_O_2_ is then converted to highly toxic ·OH through a Fenton reaction [105]. The released CDDP and generated ·OH radicals interact synergistically to induce cytotoxicity, thereby enhancing the therapeutic effect. This method offers a simple preparation process, precise therapeutic effects, and clinical significance as an anticancer strategy. The research group led by Ying developed a novel treatment strategy for osteosarcoma by combining injectable agarose hydrogels (AG) with drug-loaded FeGA-DOX nanoparticles [106,107]. Under NIR laser irradiation, the FeGA-DOX nanoparticles generate heat, inducing cellular apoptosis through hyperthermia. Simultaneously, the localized temperature increase promotes the release of FeGA-DOX within the tumor. Doxorubicin (DOX) promotes the generation of hydrogen peroxide (H_2_O_2_), while FeGA converts H_2_O_2_ to hydroxyl radicals (·OH) through a Fenton reaction under acidic conditions. Thus, the FeGA-DOX + AG treatment achieves effective tumor cell killing [107]. This innovative approach integrates the advantages of photodynamic hyperthermia and chemical drug therapy using self-oxygenating AG-coated FeGA-DOX, demonstrating potential for clinical applications. The research group led by Huang utilized MIL-100 nanoparticles as a carrier and performed surface modifications, including polydopamine (PDA) coating and NH2-PEGTK-COOH encapsulation, resulting in active Oxa@MIL-PDA-PEGTK nanoparticles [108]. In the TME, these nanoparticles release Fe^2+^ under NIR irradiation. The released Fe^2+^ can convert H_2_O_2_ into ·OH through a Fenton reaction, inducing tumor cell apoptosis and effectively suppressing tumor growth, with minimal adverse effects on the normal cells [108]. The nanoparticles also have the capability to convert light energy into heat, generating a hyperthermic effect at the tumor site in order to kill the tumor cells through photothermal therapy. This study may pave the way for designing novel nanoplatforms that respond to the tumor microenvironment, thereby enhancing the treatment outcomes for liver cancer patients in the future. The research group led by Liu synthesized Fe_3_S_4_-PEG-GOD composite nanoparticles for the treatment of pediatric tumors [109]. These nanoparticles enable the synergistic effects of photothermal therapy induced by NIR irradiation and nano-catalytic therapy. The Fe_3_S_4_ nanoparticles possess a large amount of glucose oxidase (GOD), which can catalyze the production of H_2_O_2_ for the Fenton reaction. Under NIR irradiation, the localized heating of tumor cells promotes the catalytic Fenton reaction of the nanoparticles, generating highly toxic ·OH for tumor treatment [110]. The biocompatible Fe_3_S_4_-PEG-GOD cascade nano-catalyst combines the synergistic functions of photodynamic thermal therapy and photoacoustic imaging, providing a promising therapeutic and diagnostic integrated nanoplatform for pediatric tumor treatment. The research group led by Yu synthesized Fe-PDA-EPI@FA-RBCm nanoparticles [111] (Figure 14). The high metabolic rate and abnormal blood supply in tumor cells result in an acidic environment within the tumor tissue [112]. When the nanoparticles are exposed to NIR light in the tumor cells, the polydopamine (PDA) layer on the surface of Fe-PDA-EPI@FA-RBCm nanoparticles, which contains acid dissociation groups, interacts with the acidic TME, leading to a change in the surface charge of the nanoparticles and the subsequent release of loaded iron ions (Fe^3+^). The released iron ions undergo a Fenton reaction with H_2_O_2_, generating highly reactive ·OH that ultimately induces tumor cell death [111]. Additionally, the PDA component in Fe-PDA-EPI@FA-RBCm nanoparticles can absorb NIR light and convert it into heat, raising the temperature of the surrounding environment and accelerating the progress of the Fenton reaction. The research group led by Hu synthesized Cu^2+^-doped zinc phosphate-coated Prussian blue nanoparticles (PB@Cu^2+^/ZnP NPs) [113] (Figure 15). Under near-infrared (NIR) irradiation, the PB core of these nanoparticles generates significant heat, enabling efficient photothermal therapy. This not only eradicates tumor cells, but also accelerates the degradation of intracellular H_2_O_2_, producing highly toxic ·OH and facilitating the release of the anticancer drug DOX. Simultaneously, the shell of the nanoparticles, composed of Cu^2+^-doped ZnP, gradually degrades and releases DOX and Cu^2+^ in the tumor microenvironment, which is slightly acidic. The released Cu^2+^ reacts with the intracellular antioxidant glutathione (GSH), generating Cu^+^ and further catalyzing the Fenton reaction, thereby disrupting the internal balance of the tumor cells and achieving tumor cell destruction [113]. This research proposes a new strategy for constructing a multimodal therapy, offering potential for tumor treatment. The research group led by Huang successfully constructed NIR-II-activated nanoparticles (NB/CuS@PCM NPs) using menthol (NB) as a monoterpenoid sensitizer and copper sulfide (CuS NPs) as an NIR-II photothermal agent [114,115]. In the tumor microenvironment, CuS NPs release Cu^2+^ ions under NIR irradiation and reduce them to Cu^+^ ions by consuming the intracellular antioxidant glutathione (GSH). These Cu^+^ ions participate in a Fenton-like reaction, converting intracellular H_2_O_2_ into highly toxic ·OH, further damaging the tumor cells. Moreover, under NIR irradiation, NB enhances the generation of ROS within the cells. By increasing the level of ROS, the therapeutic efficacy of the nanoparticles is enhanced, leading to increased damage to the tumor cells. This research confirms that the combination of oxidative-stress-induced damage and photothermal therapy is a potential strategy for cancer treatment [115]. The research group led by Hu developed multifunctional nanoparticles (DMTH NPs) by loading doxorubicin (DOX) onto mesoporous iron (III) terephthalate (MIL-100) and modifying them with tannic acid (TA) and hyaluronic acid (HA) [116]. In the tumor microenvironment, MIL-100(Fe) converts endogenous hydrogen peroxide (H_2_O_2_) into highly toxic ·OH through a Fenton-like reaction, assisting DOX in killing the cancer cells. Additionally, the surface modification of the nanoparticles with tannic acid (TA) confers PTT capability to the DMTH NPs. Under near-infrared laser irradiation, DMTH NPs generate a photothermal effect, thereby achieving photothermal therapy. Therefore, DMTH NPs can exert multiple effects in tumor treatment, enhancing therapeutic efficacy and enabling precise guidance through photoacoustic imaging (PAI) [116]. This study provides a simple and efficient strategy for enhancing synergistic anti-tumor effects. The research group led by Guo synthesized a cascade catalytic reaction-mediated combination therapy nanoparticle platform called ICG/Fe-LDH@PEG. It was obtained by the stepwise synthesis of PEG conjugation, ICG loading, and Fe^2+^-containing layered double hydroxide (LDH). Under NIR activation, this nanoparticle can catalyze Fenton-like reactions to generate a large amount of ·OH. Furthermore, the O^2−^ generated during NIR activation can be converted to H_2_O_2_ in the presence of superoxide dismutase (SOD) inside cells, further enhancing the subsequent Fenton-like reactions. In vitro and in vivo experiments have demonstrated that ICG/Fe-LDH@PEG nanoparticles can provide excellent therapeutic effects in tumor therapy based on Fenton reactions [117]. These nanoparticles exhibit specific therapeutic effects at the tumor site, while maintaining high biocompatibility. Therefore, ICG/Fe-LDH@PEG nanoparticles offer a well-designed therapeutic strategy for safe and effective cancer treatment. The research group led by Chen synthesized GOx@MBSA-PPy-MnO_2_ nanoparticles [118]. Under NIR irradiation, these nanoparticles exhibit excellent photothermal conversion efficiency. Through Fenton-like reactions, the MnO_2_ catalyst in the GOx@MBSA-PPy-MnO_2_ nanoparticles can catalyze the decomposition of H_2_O_2_, generating highly toxic ·OH that effectively destroys cancer cells and alleviates tumor hypoxia. In vitro and in vivo experimental results have shown that the combined therapy, triggered by photothermal therapy using GOx@MBSA-PPy-MnO_2_ nanoparticles, can inhibit cancer cell proliferation and completely suppress tumor growth [118]. Overall, this study provides a synergistic treatment approach that significantly improves therapeutic outcomes. The development of these GOx@MBSA-PPy-MnO_2_ nanoparticles holds promise as a drug-free synergistic therapy candidate for future clinical cancer treatment. The research group led by Tian synthesized biocompatible ultra-small eugenol-acid-iron-bovine-serum-albumin (EA-Fe@BSA) nanoparticles. These nanoparticles establish a rapid Fe(III)/Fe(II) conversion system by utilizing the reducing ability of endogenous hydrogen sulfide (H_2_S) to rapidly convert low-catalytic-activity Fe(III) to highly active Fe(II). This system can catalyze the rate of Fenton reactions, generating a large amount of ·OH, which disrupts the DNA, protein, and lipid structures of the cancer cells, thereby achieving tumor therapeutic effects. Furthermore, the nanoparticles exhibit strong absorption and excellent photothermal conversion efficiency under NIR irradiation, further increase the rate of the Fenton reactions through photothermal effects, and generate more (·OH), thus, enhancing the therapeutic efficacy [119]. This research, which combines endogenous H_2_S with Fenton reactions mediated by NIR, provides a novel strategy for tumor treatment. The research group led by Xiao synthesized L-buthionine sulfoximine (BSO)-modified FeS_2_ nanoparticles (BSO-FeS_2_ NPs). Under NIR irradiation, these nanoparticles exhibit excellent photothermal conversion efficiency and electron transfer capability, enabling the direct catalysis of the reaction rate in Fenton reactions and enhancing the generation of O^2−^ and (·OH), thereby improving the tumor suppression rate in vivo. The design of the BSO-FeS2 nanoparticles allows for the synergistic effects of temperature elevation, electron transfer, and ROS accumulation, thereby enhancing the effectiveness of the Fenton reactions. This research provides another feasible approach for tumor treatment [120]. The research group led by Xu utilized a simple hydrothermal and calcination method to synthesize a novel type of NiS_2_/FeS_2_ nanoparticle (NiS_2_/FeS_2_ NPs). In order to enhance their biocompatibility, the nanoparticles were further modified with polyvinylpyrrolidone (PVP), resulting in PVP-NiS_2_/FeS_2_. The PVP-NiS_2_/FeS_2_ nanoparticles exhibited the ability to inhibit tumor metastasis through multiple pathways. Under near-infrared (NIR) irradiation, the nanoparticles can induce programmed cell death, including apoptosis, ferroptosis, and pyroptosis, by generating hydroxyl radicals (·OH) via a Fenton-like reaction. Moreover, the combination of PVP-NiS_2_/FeS_2_ nanoparticles and NIR irradiation can disrupt the tumor’s immunosuppressive microenvironment and activate systemic anti-tumor immune responses, thereby eliminating metastatic tumors. This effect is partly attributed to the induction of ferroptosis and pyroptosis, which have been proven to activate tumor immunity. Furthermore, PVP-NiS_2_/FeS_2_ nanoparticles are easy to prepare and exhibit no significant toxicity towards normal organs. Therefore, PVP-NiS_2_/FeS_2_ nanoparticles possess a six-fold functionality and hold potential for imaging and treating metastatic solid tumors [121]. The research group led by Wu synthesized a multifunctional nanoparticle called Fe_3_O_4_@PPy-PEG, which consists of Fe_3_O_4_ as the core, PPy as the shell, and polyethylene glycol (PEG) [122]. Under NIR irradiation, the PPy coating exhibits photothermal effects, which not only can ablate cancer cells, but can also enhance the release of Fe^2+^/^3+^ from Fe_3_O_4_ nanoparticles. The released Fe^2+/3+^ can catalyze the Fenton reaction, generating a large amount of ·OH, which induces apoptosis in tumor cells. The PEG-modified nanoparticles demonstrate excellent stability in vivo and a prolonged circulation time in the bloodstream. These nanoparticles not only achieve effective tumor destruction, but also have no significant adverse effects on normal cells [122]. This study provides a new strategy for combining the Fenton reaction, photothermal therapy, and magnetic-guided cancer treatment, constructing an efficient and multifunctional nanoplatform for cancer therapy. The research group led by Yao synthesized a multifunctional nanoparticle called FePt/BP-PEI-FA NPS by incorporating FePt nanoparticles (FePt NPs) into ultrathin black phosphorus nanosheets (BPNs) [123]. BPNs provide efficient photothermal conversion and photodynamic effects, endowing the nanoparticles with excellent photothermal conversion efficiency and the ability to generate hydroxyl radicals (·OH) through FePt-based Fenton reactions within the TME. In vivo experiments have demonstrated the remarkable anti-tumor efficacy of FePt/BP-PEI-FA nanoparticles under NIR laser irradiation. These nanoparticles can suppress the growth of both primary and metastatic tumors, making them a promising multimodal cancer therapeutic agent [123]. The research group led by Chen constructed a nanoplatform known as PEG-PDA@Mn (PP@Mn) nanoparticles, which involved the modification of polyethylene glycol (PEG) with poly(lactic-co-glycolic acid)-polydopamine (PDA) nanoparticles. In the acidic environment of the TME, manganese ions (Mn^2+^) are released from the PP@Mn nanoparticles. Subsequently, Mn^2+^ participates in the Fenton reaction, reacting with H_2_O_2_ to generate highly reactive hydroxyl radicals (·OH) that can disrupt the cancer cells. Therefore, the PP@Mn nanoparticles induce ferroptosis through the generation of ·OH via the catalytic Fenton reaction. In vitro studies have shown that the PP@Mn nanoparticles, in combination with photothermal therapy, generate a large amount of ·OH, leading to cancer cell death. In in vivo experiments, the PP@Mn nanoparticles provide valuable tumor visualization information through MRI. Additionally, the PP@Mn nanoparticles significantly inhibit tumor growth and exhibit satisfactory biocompatibility [124]. The PP@Mn nanoplatform represents a novel theranostic system with promising applications in the field of gastric cancer treatment. The research group led by Fang synthesized PEG-modified CuMo_2_S_3_ nanoparticles (CMS NPs) using a hydrothermal method. These nanoparticles exhibit a dandelion-like nanostructure and generate high temperatures and reactive oxygen species (ROS) under NIR irradiation. The presence of multivalent elements (Cu^+/2+^, Mo^4+/6+^) in the nanoparticles allows them to undergo a Fenton-like reaction, with excessive H_2_O_2_ present in the tumor microenvironment (TME), thereby generating hydroxyl radicals (·OH) and enhancing the therapeutic effect. In vitro experiments have demonstrated the excellent photothermal conversion efficiency and particle morphological stability of CMS NPs. Furthermore, CMS NPs exhibit significant enhancement in anticancer efficacy, good biocompatibility, and large thermal effects under NIR laser irradiation [125]. These findings highlight the promising potential of CMS NPs in cancer treatment and suggest a favorable outlook for their clinical applications.

Using light-responsive nanoparticles to catalyze the Fenton reaction for the treatment of malignant tumors is a promising therapeutic strategy (Table 1.). This approach utilizes specifically designed nanoparticles that can generate ROS under light illumination, inducing the Fenton reaction in order to destroy tumor cells. This treatment method offers several advantages. Firstly, controlling the release of ROS through light responsiveness allows for localized and targeted therapy, minimizing damage to normal tissues. Secondly, the highly reactive oxygen species generated by the Fenton reaction possess strong oxidative properties, capable of damaging the key structures in tumor cells, such as DNA, proteins, and lipids, thereby inducing apoptosis and necrosis in tumor cells. Additionally, the surface of the nanoparticles can be functionalized in order to achieve selective enrichment in tumor cells, further enhancing therapeutic efficacy. However, this treatment strategy still faces some challenges, including the synthesis and stability of the nanoparticles, the control of light conditions, and ensuring the biocompatibility and safety in the human body, all of which require further research and optimization. In conclusion, the therapeutic strategy based on light-responsive nanoparticles catalyzing the Fenton reaction provides a new avenue and potential for the treatment of malignant tumors.

## 3. Conclusions

In summary, the application of the Fenton reaction in the cancer microenvironment holds immense potential as a therapeutic strategy. The unique characteristics of the tumor microenvironment, such as the elevated levels of H_2_O_2_ and the presence of transition metal ions, make it an ideal target for the Fenton reaction. By utilizing these components, the Fenton reaction can generate highly reactive ·OH, which has potent oxidizing capabilities, leading to the oxidative damage and subsequent death of tumor cells. One of the major advantages of the Fenton-reaction-based therapy is its high efficiency in selectively targeting tumor cells while minimizing damage to healthy tissues. The ability to precisely control the release of ROS through the modulation of the reaction conditions allows for localized treatment and reduced side effects. Moreover, the Fenton reaction offers a multifaceted approach to tumor cell destruction, targeting critical cellular components such as DNA, proteins, and lipids. In addition to its efficacy, the Fenton reaction can be combined with other therapeutic modalities, such as chemotherapy, photodynamic therapy, or immunotherapy, in order to achieve synergistic effects and enhance the overall treatment outcomes. The versatility of the Fenton reaction opens up possibilities for combinatorial approaches in cancer therapy, enabling a personalized and tailored treatment strategy. However, several challenges remain in the application of the Fenton reaction for cancer treatment. The synthesis and stability of the nanoparticles and the precise control of the reaction conditions need further refinement. Additionally, ensuring the biocompatibility and safety of the nanoparticles and understanding their biodistribution in vivo are essential considerations for clinical translation. In conclusion, the Fenton-reaction-based therapy presents a promising avenue for cancer treatment. Continued research and optimization in nanoparticle synthesis, reaction control, and safety assessment will further advance this field and bring us closer to harnessing the full potential of the Fenton reaction in combatting malignant tumors.

## Figures and Tables

**Table 1:** A Comparative Analysis of Advantages and Disadvantages of the Polymer Nanoparticle-Catalyzed Fenton Reaction for Malignant Tumor Treatment and Application of the Photo-thermal-Enhanced Fenton-like Reaction Using Nanoparticles in the Tumor Microenvironment.

	Polymer Nanoparticle-Catalyzed Fenton Reaction for Malignant Tumor Treatment	Application of Photothermal-Enhanced Fenton-like Reaction Using Nanoparticles in the Tumor Microenvironment
Advantage	High efficacy, targeted therapy, and biocompatibility.	Greater controllability, reduced harm to healthy tissues, and the ability to achieve more precise treatment.
Disadvantage	Compared to other nanoparticles, the efficiency of polymer nanoparticles in catalyzing Fenton-like reactions may be relatively low. The stability of polymer nanoparticles could be challenged, potentially affecting their long-term application.	The synthesis and stability of nanoparticles, control of light conditions, and ensuring biocompatibility and safety in the human body.

## Data Availability

All data and materials in this study are included in the published article.
